# The Biological Activities of Mango Seed Fractions and Its Hepatoprotective Effects on Alcoholic Liver Disease and Modulation of Intestinal Flora

**DOI:** 10.3390/foods15071116

**Published:** 2026-03-24

**Authors:** Zaixiang Lou, Xu Cheng, Zejun Pei, Caihua Liu, Zhengjie Zhu, Yuemei Liao, Huili Huang, Rui Huang, Yaqin Li

**Affiliations:** 1 Center for Food Nutrition and Functional Food Engineering, School of Food Science and Technology, Jiangnan University, Wuxi 214122, China; chengxu1102@outlook.com (X.C.); pei-zj@126.com (Z.P.); huangrui020118@163.com (R.H.); 2 Guangxi Key Laboratory of Biology for Mango, Baise University, Baise 533000, China; liucaihua519@126.com (C.L.); zhuzhjie@163.com (Z.Z.); llss19961997@163.com (Y.L.); 3 University Engineering Research Center for Preservation and Comprehensive Utilization of Subtropical Characteristic Agricultural Products in Guangxi, Baise 533000, China; 4 Baise Modern Agriculture Technology Research &Extension Center, Baise 533612, China; 13687880566@163.com; 5 School of Intelligent Manufacturing, Jiangsu Agri-Animal Husbandry Vocational College, Taizhou 225300, China; liyaqin2000@126.com

**Keywords:** mango, hypolipidemic, intestinal flora, UPLC-MS/MS

## Abstract

In this study, the active components in the seed of *Mangifera indica* L. were isolated, the main chemical components were identified, and then their antioxidant activities and their effects on liver injury and intestinal microbiota were evaluated. The results showed that all the components of mango column chromatography exhibited antioxidant activity. F2 had the lowest IC_50_ value of 93.61 μg/mL and exhibited the strongest DPPH radical scavenging activity. Given its superior overall antioxidant activity, F2 was selected for further compositional analysis and activity evaluation. UPLC-MS/MS analysis showed that the isolated components of mango F2 contained 11 active ingredients, including mangiferin, gallic acid and quercetin. The results showed that specific mango fractions significantly reduced serum alanine aminotransferase (ALT) and aspartate aminotransferase (AST) levels, and showed a protective effect on liver injury induced by alcohol. rRNA sequencing analysis showed that high alcohol intake could reduce the species diversity of intestinal microbiota in mice, and mango fractions could effectively alleviate this phenomenon. High alcohol intake decreases the relative abundance of *Bacteroidota* and increases the abundance of Bacillota and *Thermodesulfobacteriota* phyla. The high-dose mango group alleviated the above changes, which was manifested by an increase in the relative abundance of *Bacteroidota* and *Thermodesulfobacteriota* bacteria. The relative abundance of families such as *Muribaculaceae* in the high-dose mango group decreased compared to the model group. This study provides a scientific basis for the analysis and high-value utilization of mango components, and provides a new alternative for protecting against alcoholic liver injury and regulating intestinal microbiota.

## 1. Introduction

Alcoholic liver disease (ALD) is one of the most common liver diseases worldwide, and long-term excessive alcohol consumption can lead to liver metabolic disorders, increased oxidative stress and increased inflammatory response, which in turn can lead to fatty liver, hepatitis, liver fibrosis and even liver cirrhosis. Studies have shown that alcohol and its metabolites (such as acetaldehyde) can disrupt the redox balance in hepatocytes by inducing the production of reactive oxygen species (ROS), thereby accelerating hepatocyte damage [1]. In addition, alcohol intake can also alter the composition of the gut microbiota, disrupt the intestinal barrier function, and promote the entry of intestinal endotoxins (such as lipopolysaccharides, LPS) into the portal vein circulation, further exacerbating the inflammatory response of the liver [2]. Therefore, it is of great significance to explore natural active ingredients with antioxidant, anti-inflammatory, and intestinal microbiota modulation effects for the prevention and treatment of alcoholic liver injury.

As a widely cultivated tropical fruit, mango (*Mangifera indica* L.) has high nutritional and medicinal value due to its flesh rich in vitamins, polyphenolic compounds and dietary fiber. During mango processing, seeds account for 20–35% of the fresh fruit weight. Traditional disposal by discarding not only causes resource waste but also imposes environmental pressures (such as mold contamination). In recent years, mango seeds have attracted attention due to their rich bioactive components such as mangiferin and quercetin. Studies have shown that mango seed extract has a variety of physiological activities, such as antioxidant, anti-inflammatory and hypolipidemic effects [3,4]. However, the isolation and identification of specific active ingredients in mango seeds and their mechanism in the protective effect of alcoholic liver injury are still limited. Therefore, the systematic analysis of the active ingredients in mango seeds and the exploration of their regulatory effects on alcoholic liver injury and intestinal microbiota [5] will not only help to expand the high-value utilization of mango resources, but also provide a theoretical basis for the development of new hepatoprotective functional foods or drugs.

Ultra-performance liquid chromatography-tandem mass spectrometry (UPLC-Q-TOF-MS/MS) has become an important tool for the identification of chemical components of natural products due to its advantages of high resolution (≥10,000), high mass accuracy (≤5) ppm and rapid analysis. Through this technology, the active components such as polyphenols and flavonoids in mango seeds can be accurately identified, and their antioxidant activities can be evaluated in combination with in vitro antioxidant experiments (such as DPPH and ABTS free radical scavenging capacity determination). In addition, the establishment of an animal model of alcoholic liver injury can further verify the hepatoprotective effect of mango’s active ingredients, including the effects on serum alanine aminotransferase (ALT) and aspartate aminotransferase (AST) levels and liver indicators. At the same time, the intestinal microbiota plays an important role in the occurrence and development of alcoholic liver injury. The application of high-throughput 16S rRNA sequencing technology can comprehensively analyze the regulatory effect of mango’s active ingredients on alcohol-induced intestinal microbiota disorders, such as the diversity of microflora, the abundance of dominant bacteria (*Bacteroidota*, *Firmicutes*, etc.) and specific probiotics (such as *Lactobacillus* and *Bifidobacteria*).

In this study, column chromatography was used to systematically separate the active components in mango seeds, and the main chemical components were identified based on high-resolution UPLC-Q-TOF-MS/MS. Comparison were then made with standard compounds. On this basis, the most active component was screened out by in vitro antioxidant experiments, and its protective effect on mouse models of alcoholic liver injury and its regulatory effect on intestinal microbiota were further explored. This study not only provides a scientific basis for the in-depth analysis of the active ingredients of mango seeds, but also lays a theoretical foundation for its application in the prevention of alcoholic liver injury and the regulation of intestinal microecology, which aligns with the trend of “high-value utilization of agricultural by-products”.

## 2. Materials and Methods

### 2.1. Materials and Reagents

The mango fruit was produced in Guangxi, and the seed was extracted, freeze-dried, and crushed. The standards, including shikimic acid (97%), gallic acid (97%), catechinic acid (99%), mangiferin (98%), p-Coumaric acid (99%), kaempferol hyperoside (98%), puerarin puemfin (98%), protocatechuic acid (97%), ellagic acid (97%), and quereetin (98%) were bought at Sigma (Shanghai, China). The chemical reagents were analytically pure. Alanine aminotransferase (ALT) test kits and aspartate aminotransferase (AST) test kits were provided by Nanjing Jiancheng Institute of Bioengineering (Nanjing, China).

### 2.2. Extraction and Separation of Mango Components

Mango seed powder (100 g) was weighed, 2000 mL of 70% ethanol (1:20, *w*/*v*) was added, and ultrasonic extraction (40 kHz, 50 °C, 30 min) was repeated three times. Then the extraction solution was combined and concentrated under reduced pressure. The pretreated AB-8 resin was wet-packed into a column (Φ25 × 500 mm). Two bed volume (BV) 10.00 mg/mL of aqueous mango extract was loaded onto the resin column at a loading flow rate of 1.0 BV/h. After adsorption equilibration, 12.0 BV, 4.0 BV, 6.0 BV and 4.0 BV were eluted by water and 20%, 40% and 70% (*v*/*v*) ethanol gradients, respectively, with an elution flow rate of 1.0 BV/h. The 20%, 40% and 70% ethanol-eluted phases are named F1, F2 and F3, respectively.

### 2.3. Ultra-Performance Liquid Chromatography-Tandem Quadrupole Time-of-Flight Mass Spectrometry (UPLC-Q-TOF-MS)

Mango component samples (10.00 mg) were dissolved in 10 mL of methanol. Phenolic compounds were identified by UPLC-Q-TOF-MS [6]. A BEH C18 column (2.1 × 50 mm Agilent, Santa Clara, CA, USA) was used for chromatographic separation in a 35 °C column box and quantified by four-stage pole flight mass spectrometry. Ionization took place in negative ion mode. The injection volume of the sample was 5 μL, while the column temperature was 40 °C, and the detection wavelength was 280 nm. Mobile phase A: 0.05% (*v*/*v*) formic acid; Mobile phase B: 100% acetonitrile. Data were processed using Waters MassLynx 4.2.

### 2.4. Antioxidant Activity Assay

#### 2.4.1. DPPH Free Radical Scavenging Capacity

DPPH free radical clearing (1,1-Diphenyl-2-picrylhydrazyl DPPH) ability was determined according to the method of He et al. [7]. The sample solution (2 mL) and 2 mL of 0.2 mmol/L DPPH solution were added to the test tube, mixed well, and reacted in the dark for 30 min. The mixture was then centrifuged at 3000 r/min for 10 min, and the OD 517 nm value was measured and recorded as A_s_. The blank group with absolute ethanol instead of DPPH solution was denoted as A_0_, and the solvent used to prepare the samples was used as the control group. DPPH free radical scavenging rate (%) was calculated and repeated three times per group.

#### 2.4.2. Determination of Hydroxyl Radical Scavenging Capacity

For the determination of hydroxyl radical scavenging ability [8], 1 mL of 2.5 mmol/L pH florin and 1 mL of PBS was mixed. A further amount of 1 mL of 2.5 mmol/L FeSO_4_ and 1 mL of 20 mmol/L H_2_O_2_ were added. The mixture was then placed under a 37 °C water bath for 90 min and then measured at 526 nm while the OD value was denoted A_1_. The sample was replaced with 1 mL of water as the blank group, which was denoted as A_2_ (background absorbance without), and 1 mL of water was used instead of H_2_O_2_ as the control group, denoted as A_0_ (maximum radical activity). Hydroxyl radical scavenging rate (%) was calculated and repeated three times per group.

#### 2.4.3. Determination of Superoxide Anion Radical Scavenging Ability

For the determination of superoxide anion radical scavenging ability, we referred to the method of Yu et al. [9] The sample solution (0.5 mL) and 1.5 mL of Tris-HCl solution were added to a tube, and mixed well in a water bath at 25 °C for 20 min. Then, 200 μL of 25 mmol/L catrogallone was added, and 250 μL HCl was added to stop the reaction. Then the mixture was centrifuged at 3000 r/min for 10 min, and the OD_325_ was measured and recorded as A_sample_. The superoxide anion radical scavenging rate (%) was calculated and each group was repeated three times.

### 2.5. Animals and Treatments

#### 2.5.1. Grouping and Feeding of Mice

The mice were housed at the Experimental Animal Center of Jiangnan University (SPF grade), with a temperature of 23 ± 3 °C, a relative humidity of 55 ± 15%, and a regular 12 h alternating cycle of light and dark. The animal experiment was approved by the Animal Welfare and Ethics Committee of Jiangnan University: JN. No20240415c1200830. After one week of adaptive rearing, the mice were randomly divided into six groups, with 8 mice per group (n = 8): the blank control group (CON), the model control group (MOD), the positive control group (POS), the low-dose group (MF-L), the medium-dose group (MF-M), and the high-dose group (MF-H).

The first seven days consisted of the adaptation period. From the 8th to the 35th day, distilled water was administered by gavage to the CON group and the MOD group every day. Biphenyl diester (150 mg/kg BW) was administered by gavage to the POS group, and the MF-L group, MF-M group and MF-H group were administered with mango components (10.00 mg/mL aqueous solution of mango active fraction F2 extract) [10]. The dosage for each administration was 10 mg/kg BW. Six hours after the last administration, the CON group was intragastrically treated with distilled water, while the remaining groups of mice were intragastrically treated with 50% ethanol at a dose of 12 mL/kg BW [11].

#### 2.5.2. Determination of Serum Biochemical Indicators and Liver Index

After the last gavage was completed, the mice were fasted overnight and then anesthetized with a mixture of 1–1.5% isoflurane and oxygen. After the mice were completely anesthetized, blood was collected from the eyeballs. Subsequently, the mice were killed by neck removal. The blood samples were centrifuged at 3500 r/min at 4 °C for 15 min to obtain mouse serum, and the levels of AST and ALT in the serum were determined. The liver tissue of the mouse was removed and weighed to calculate the liver index. The calculation formula is as follows:

Liver index = Liver mass/(mouse body weight) × 100%.

#### 2.5.3. Sequencing of Mouse Intestinal Flora

DNA was extracted from fecal samples, 1% agarose gel electrophoresis was tested, and qualified samples were sent to Shanghai Meiji Biomedical Technology Co., Ltd. (Shanghai, China). for amplification sequence. The primers are:

338F: ACTCCTACGGGAGGCAGCAG; 806R: GGACTACHVGGGTWTCTAAT. Fecal microbiota was sequenced using the Illumina MiSeq sequencing platform (v4.1.0), resulting in 16S rRNA data.

### 2.6. Statistical Analysis

All the experiments were performed in triplicates. The data represents the mean of the triplicate values. SPSS 23.0 statistical software was used for data analysis. One-way ANOVA was used for comparison between multiple groups, and *p* < 0.05 was statistically significant.

## 3. Results and Discussion

### 3.1. Antioxidant Activity of the Purified Components by Column Chromatography

#### 3.1.1. Superoxide Anion Radical Scavenging Activity of Mango Isolate Components

Superoxide anion can produce hydrogen peroxide and hydroxyl radicals through disproportionation or other reactions; superoxide anion radicals exist for a long time and can move over long distances. As a precursor to reactive oxygen species (ROS) and other ROSs, superoxide anions are often used as markers to detect early reactive oxygen species formation [12]. Therefore, it is of great significance to study the scavenging of superoxide anions. It was found that the scavenging rate of superoxide anion changed with the alteration in the concentration of each separated component. Three fractions (F1, F2, F3) were obtained via AB-8 resin column chromatography with gradient elution (20%, 40%, 70% ethanol), and their antioxidant activities were evaluated by three in vitro assays. At a concentration of 200 μg/mL, TBHQ and three mango components (F1, F2, F3) were 78.86, 17.61, 76.30, and 27.53% (Table 1). The F2 component could effectively scavenge superoxide anion radicals. When the concentration of F2 was 200 μg/mL, the clearance rate of superoxide anion reached 76.30%. F2 had the strongest scavenging activity among the three components, and for all concentrations. F2 had the best inhibitory activity, followed by F3 and F1. At 400 μg/mL, TBHQ, F2, F1 and F3 had scavenging activities of 86.32%, 81.97%, 23.83%, and 39.61%, respectively. The results show that F2 could effectively inhibit the oxidation reaction caused by superoxide anion radicals, indicating that F2 had strong chain-interruption antioxidant activity.

#### 3.1.2. Hydroxyl Radical Scavenging Activity

Hydroxyl radicals are important initiators of liposomal peroxidation. A hydroxyl radical is a highly oxidizing free radical: the higher the scavenging power of hydroxyl radicals, the stronger the antioxidant capacity [13,14]. Table 2 shows the scavenging power of each isolated component of mango for hydroxyl radicals. The components showed concentration-dependent scavenging activity (Table 2). At 800 μg/mL, compared to TBHQ (clearance rate of 68.72%) F2 showed stronger hydroxyl radical scavenging activity (71.30%).

#### 3.1.3. DPPH Free Radical Scavenging Activity

A DPPH free radical is a very stable free radical [15,16,17,18]. The antioxidant capacity of mango was indicated by measuring the scavenging capacity of the isolated components of mango. The stronger the DPPH radical scavenging capacity, the greater the antioxidant capacity of the component [16,17].

DPPH clearance capacity is often expressed as IC_50_. The results showed that the IC_50_ of F2, TBHQ, F1 and F3 were 93.61, 95.63, 861.30 and 629.19 μg/mL, respectively (Table 3). The clearance capacity of F2 is stronger than that of TBHQ. Among the various mango isolates, F2 had the lowest IC_50_ and exhibited the strongest DPPH radical scavenging activity.

The normal physiological metabolism of the human body will produce reactive oxygen species (RSO), which will promote the process of cell differentiation and apoptosis at low levels and cause damage to cells at high concentrations, resulting in mutations, apoptosis and necrosis, and further cause hypertension, diabetes and other diseases. At the same time, when the body undergoes oxidative stress, oxygen free radicals are also produced and damage the central nervous system [19,20,21]. Therefore, researchers usually use superoxide radicals and hydroxyl radicals as indicators to study antioxidant capacity [19,20,21,22]. Scientific studies have confirmed that mango components have significant antioxidant effects [3,23], similar to our findings. The results show that F2 had the strongest antioxidant activity, so F2 was selected to study its effect on liver injury and intestinal microbiota.

### 3.2. Composition Analysis

**Figure 1:**
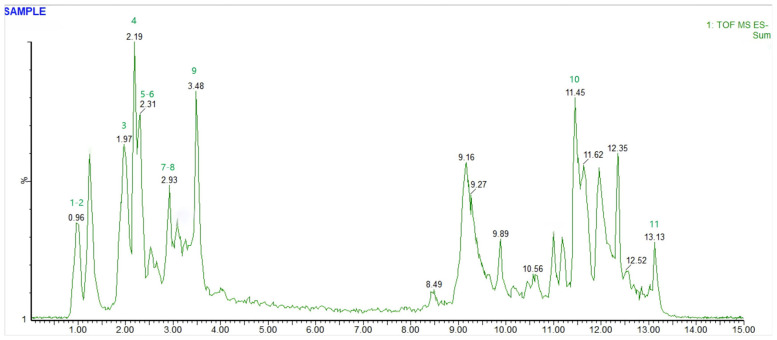
UPLC-Q-TOF-MS total ion chromatograms (TIC) of mango fraction F2 (Numbers 1–11 means identification of isolated components of mango in Table 4.).

In order to facilitate the analysis of the chemical composition of F2, a component isolated from mango kernels, the chemical composition of the mango component was retrieved based on CNKI, GoogleScholar, SciFinder and other databases, and then the chemical composition information of the corresponding components was obtained through ChemSpider, ChemBook, MassBank, PubMed, etc., including the name, molecular formula, precise molecular weight and other information so as to establish a database of the chemical composition of the mango component. On this basis, a total of 11 compounds were identified by comparing them with the standard substance and combining the quasimolecular ion peaks and secondary fragmentation information in the negative ion mode of UPLC-Q-TOF-MS/MS (Table 4). The total ion flow of the mango component F2 is shown in Figure 1. Compound **2** has an excimer ion peak of *m*/*z* 169.01 [M–H]^−^, and the molecular formula is C_7_H_6_O_5_ in negative ion mode. In the mass spectrometry secondary cleavage diagram, the *m*/*z* 125.02 [M–H–CO_2_]^−^ ion signal peak generated by the [M–H]^−^ ion peak was lost in one molecule of CO_2_. Compound **2** was identified to be gallic acid by comparison with the reference substance. The quasimolecular ion peak of compound **4** in negative ion mode was *m*/*z* 421.08 [M–H]^−^, the molecular formula was C_19_H_18_O_ll_, and the signal peaks of *m*/*z* 331.04 and 301.03 were observed in the mass spectrometry secondary cleavage map. At the ion peak, [M–H]^−^ glycan ring cleavage occurs. Compound **4** was identified to be mangiferin by comparison with the reference standard.

### 3.3. Effects of Components of Mango on Serum Biochemical Indicators of Mice

**Figure 2:**
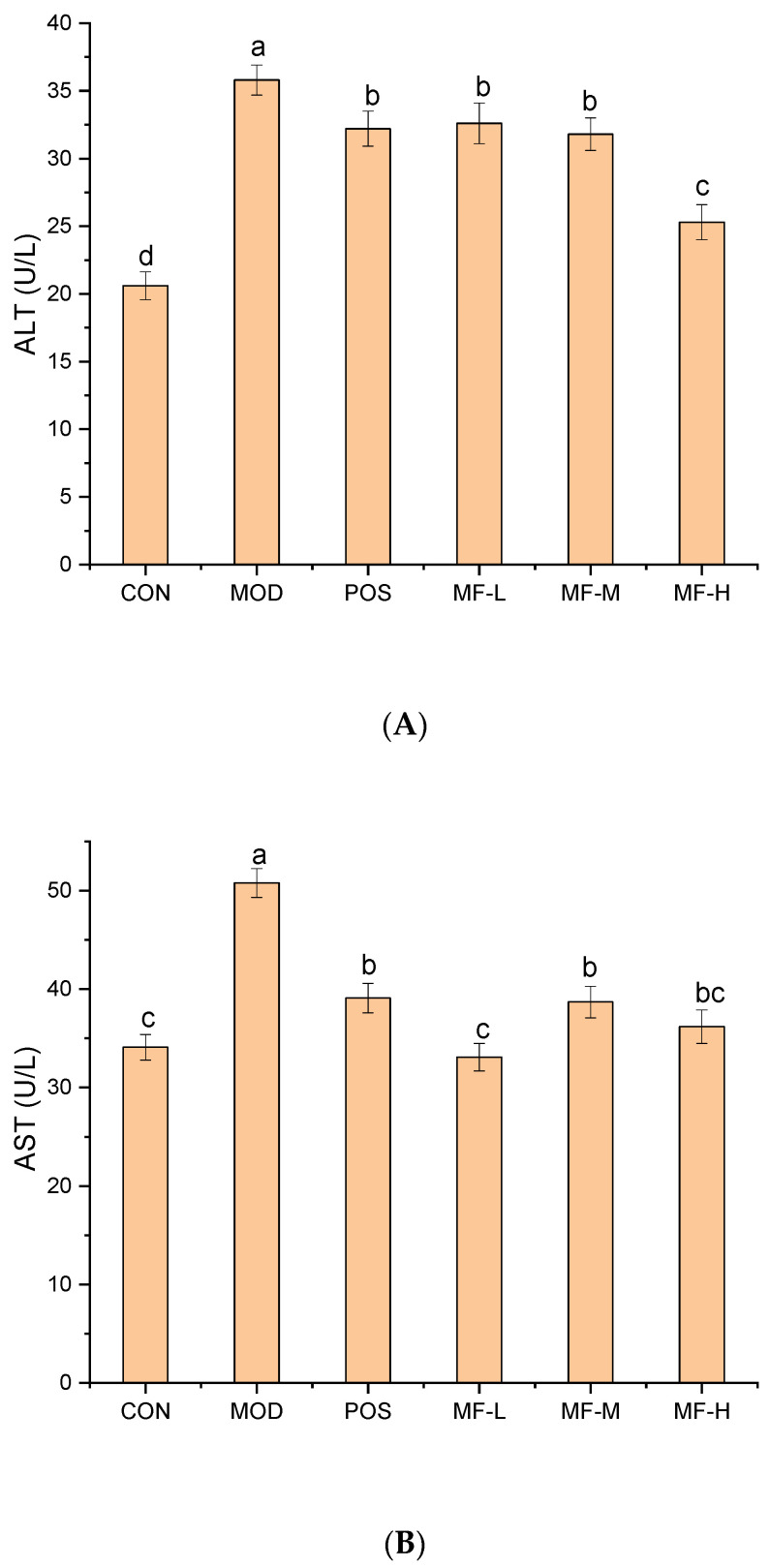
The effect of MF on the levels of ALT (**A**) and AST (**B**) in mouse serum. Significant differences among samples are indicated by different lowercase letters (*p* < 0.05).

To explore the protective effect of mango separation fraction (MF) F2 on alcoholic liver injury, the levels of serum biochemical markers ALT and AST were determined. Under normal circumstances, ALT and AST are mainly present in the liver. However, when excessive alcohol intake occurs, the liver cell membranes are damaged, and ALT and AST are released into the bloodstream, leading to abnormal increases in the content of ALT and AST in the serum [24]. The ALT and AST of mice in each group are shown in Figure 2. Compared with the CON group, the activities of ALT and AST in the serum of mice in the alcohol-administered MOD group were significantly increased (*p* < 0.05), with the ALT activity reaching 38.36 U/L and the AST activity reaching 50.80 U/L, indicating that the MOD for acute alcoholic liver injury in mice was successfully constructed. Compared with the MOD group, the activities of ALT and AST in the three experimental groups of MF-L, MF-M, and MF-H decreased significantly (*p* < 0.05), indicating that MF can effectively resist the damage of alcohol intake to liver cells, maintain the integrity and permeability of the cell membrane, and reduce the leakage of ALT and AST. It has a significant protective effect on alcoholic liver injury.

### 3.4. Effects of MF on Liver Indices in Mice

**Figure 3:**
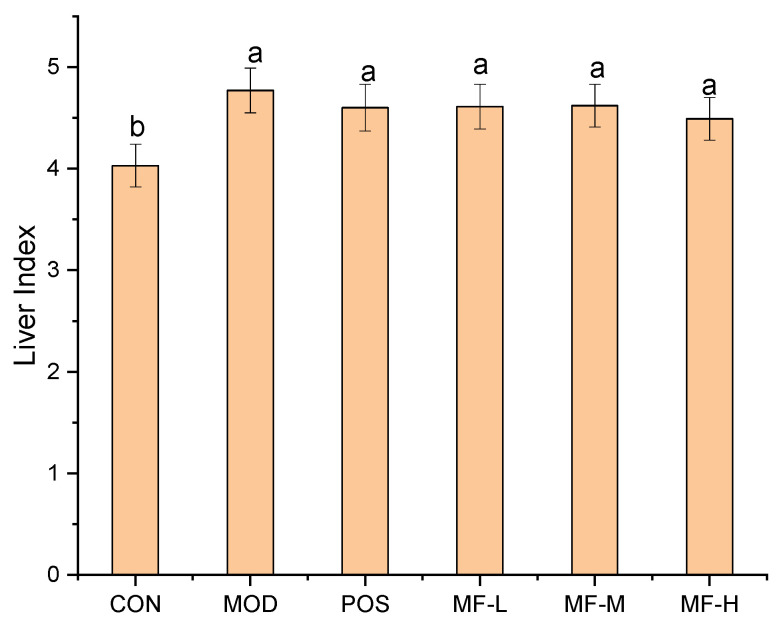
Liver index. Significant differences among samples are indicated by different lowercase letters (*p* < 0.05).

The liver indices of the mice are shown in Figure 3. Compared with the CON group, the liver indices of the mice in the MOD group significantly increased by 0.73% (*p* < 0.05), preliminarily indicating that acute intragastric alcohol administration caused liver damage to the mice, and the experimental modeling was successful. The liver indices of the POS group and the MF-L, MF-M, and MF-H groups were all lower than those of the MOD group, indicating that MF alleviated acute alcoholic liver injury in mice.

### 3.5. Effect on the Composition of Intestinal Microbiota

#### 3.5.1. Effects of Mango Component F2 on the Diversity of Intestinal Microbiota of Mice

**Figure 4:**
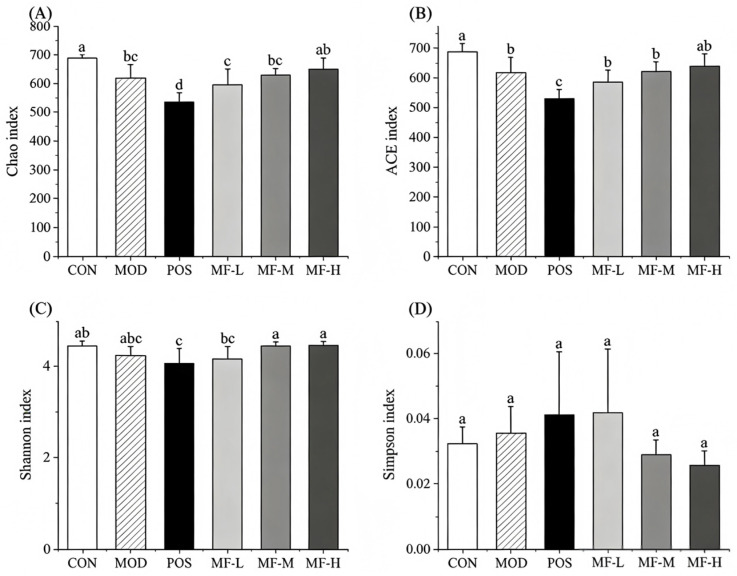
α Diversity index of intestinal microbiota in mice: (**A**) Chao index; (**B**) ACE index; (**C**) Shannon index; (**D**) Simpson index. Significant differences among samples are indicated by different lowercase letters (*p* < 0.05).

The diversity of intestinal microbiota between groups was analyzed by α diversity index. The Chao and ACE indices reflect the OTU level of species richness and uniformity. The Shannon and Simpson indices reflect the degree of difference in species diversity. As shown in Figure 4, the Chao and ACE indices in the model (MOD) group significantly decreased compared with the control group (CON, *p* < 0.05), indicating that the alcohol administration had a tendency to reduce the number of species. Compared with the model group, the Chao and ACE indices of intestinal microbiota in the mango fraction F2 high-dose (MF-H) group were slightly higher than those in the model group. The Shannon and Simpson indices differed slightly among the groups, suggesting that alcohol had little effect on the diversity of the gut microbiota species. The Shannon index of the MOD group was significantly lower than that of the CON group (*p* < 0.05). The Simpson index was not statistically significant. The Shannon index of the MF-M and MF-H groups showed an upward trend compared with that of the MOD group, but it was not statistically significant. High alcohol intake decreases the diversity of the gut microbiota species in mice, and the mango component can effectively alleviate this phenomenon.

#### 3.5.2. Effect of Mango Components on the Composition of Intestinal Microbiota in Mice

**Figure 5:**
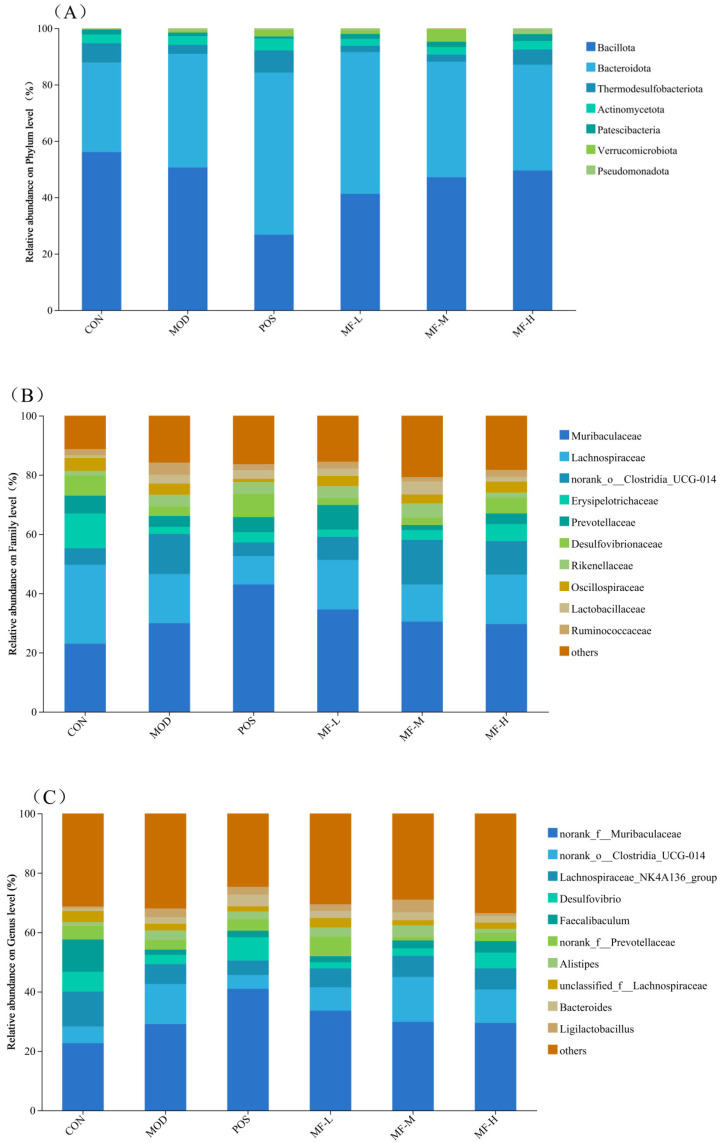
Relative abundance of intestinal microbiota in mice: (**A**) gate level; (**B**) sectional level; (**C**) genus level.

In order to further explore the effect of mango component F2 on the composition of intestinal microbiota in mice, the composition of intestinal microbiota at different levels was detected, and the top 10 species were displayed in the form of stacked histograms. Their effects on the composition of intestinal microbiota in mice were studied at the phylum, family and genus levels, respectively. Figure 5A shows that the dominant species composition at the phylum level mainly includes *Bacillota phylum*, *Bacteroidota* and *Thermodesulfobacteriota*. Usually, the abundance of *Bacteroidota phylum* are important indicators of intestinal microbiota health, and can be used to define dysbiosis. High alcohol levels increase the relative abundance of Bacteroidota and decreases the abundance of Bacillota and Thermodesulfobacteriota phyla. The high-dose mango group alleviated the above changes to a certain extent, which was manifested by an increase in the relative abundance of *Bacteroidetes* and *Thermodesulfur* bacteria. Compared with the CON group, the relative abundance of Bacillota phyla in the MOD group decreased from 56.06% to 50.62%, the relative abundance of Bacteroidota increased from 31.78% to 40.31%, and the relative abundance of *Pyrrhobacterobacterium* decreased from 6.85% to 3.20%. Compared with the CON group, the relative abundance of Bacillota did not increase, the relative abundance of Bacteroidota decreased to 37.53%, and the relative abundance of *Desthiobacter thermogenes* increased to 5.38%.

At the level of family classification, Figure 5B shows an increase in the high alcohol group compared to the CON group. The relative abundance of the *Muribaculaceae* family increased from 23.03% to 29.95% in the high-dose mango group. The relative abundance of the family decreased compared to the MOD group, and the relative abundance of the *norank_o__Clostridia_UCG-014* family varies similarly.

#### 3.5.3. Comparative Analysis of Intestinal Flora Samples

**Figure 6:**
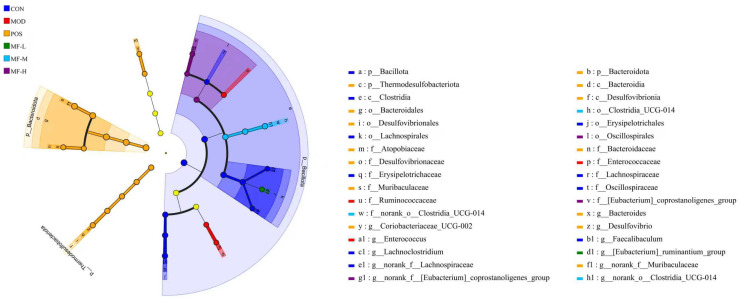
Phytoclade diagram of the dominant species composition of the intestinal microbiota of mice at different taxonomic levels; p: phylum level; c: class level; o: mesh level; f: section level; g: genus level.

**Figure 7:**
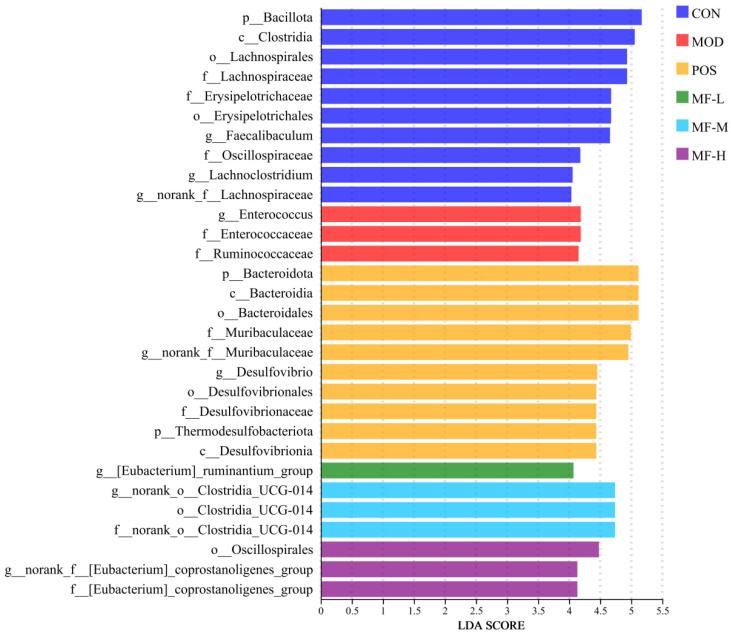
Histogram of LDA value distribution of dominant species in mouse gut microbiota.

The test results of the intestinal flora of the samples were compared through linear discriminant analysis effect size (LEfSe), thereby evaluating the differences in microbial communities between groups and obtaining the dominant species of each group. In the evolutionary branch diagram (Figure 6), the taxonomic levels from phylum to genus are represented by circles from inside to outside, and different species at the corresponding levels are represented by circles at each taxonomic level. The relative abundance of species is reflected by the diameter of the circles, and the color of the corresponding group is displayed when the abundance of a species is the highest in the group. The bar chart of LDA value distribution in Figure 7 shows the dominant species with LDA score > 4 in each group. The results showed that the microbial communities with LDA > 4 in the CON, MOD, POS, MF-L, MF-M and MF-H groups were 10 species, three species, 10 species, one species, three species, and three species, respectively. The dominant species of the CON group are mainly distributed in the *Bacillota phylum*, *Clostridia* class and *Lachnospirales* family. The alcohol intake led to the *Enterococcus* genus, *Enterococcaceae* family and *Ruminococcaceae* family becoming significantly different between the MOD group and other groups. The significantly differentiated communities in the POS group were the *Desulfovibrionia* class, the *Thermodesulfobacteriota phylum*, the *Desulfovibrionaceae* family, the *Desulfovibrionales* order, and the *Desulfovibrio* genus. In the MF-L group, the genus *[Eubacterium]_ruminantium*_group is the differential group, and *Clostridia_UCG-014SOM* is the dominant species in the MF-M group. The dominant species in the MF-H group include the *Oscillospirales* order and the *[Eubacterium]_coprostanoligenes*_group family. The components of mangoes help regulate the intestinal flora [25,26,27,28], and is conducive to the growth of beneficial intestinal flora.

## 4. Conclusions

Among the isolated components of mango, F2 had the strongest hydroxyl radical scavenging activity, DPPH free radical scavenging activity, and superoxide anion free radical scavenging activity, indicating that F2 had the strongest antioxidant activity. The results of liquid chromatography-mass spectrometry analysis showed that F2 contained 11 active components such as mangiferin and gallic acid. Animal experiments have shown that F2, the isolated component of mango, can significantly reduce serum ALT and AST levels, and display a protective effect on liver injury. The mechanism may be related to the regulation of intestinal microbiota structure. The mango isolate F2 significantly improved the diversity and composition of intestinal microbiota in mice. This study provides a scientific basis for the development and high-value utilization of mango functional components. Future studies can further explore its synergistic effect with other hepatoprotective components to develop more potential functional foods or drugs and provide new strategies for the prevention and treatment of alcoholic liver disease.

## Figures and Tables

**Table 1:** Superoxide anion radical scavenging activity of mango fractions. Significant differences among samples are indicated by different lowercase letters (*p* < 0.05).

Concentration (μg/mL)	TBHQ	F1	F2	F3
100	52 ± 3 c	8 ± 3 a	48 ± 3 c	12 ± 2 b
200	79 ± 2 c	18 ± 3 a	76 ± 4 c	28 ± 4 b
400	86 ± 4 d	24 ± 3 a	82 ± 2 c	40 ± 3 b

**Table 2:** Hydroxyl radical scavenging activity of mango fractions. Significant differences among samples are indicated by different lowercase letters (*p* < 0.05).

Concentration (μg/mL)	TBHQ	F1	F2	F3
200	21 ± 1 c	8 ± 2 a	21 ± 2 c	9 ± 2 a
400	46 ± 3 c	14 ± 3 a	44 ± 2 c	13 ± 3 a
800	69 ± 4 c	22 ± 3 a	71 ± 4 c	27 ± 4 b

**Table 3:** DPPH free radical scavenging activity of the isolated components of mango. Significant differences among samples are indicated by different lowercase letters (*p* < 0.05).

Component	IC_50_ (μg/mL)
F1	861 ± 2 c
F2	94 ± 4 a
F3	629 ± 3 b
TBHQ	96 ± 4 a

**Table 4:** Composition analysis and identification of isolated components of mango.

Peak Number	RT	Identified Compound	[M–H]	Molecular Formula	Main Fragments
1	0.95	Shikimic acid	173.04	C_7_H_10_O_5_	155.03 (M-H-H_2_O), 137.02 (M-H-2H_2_O), 119.01
2	1.24	Gallic acid	169.01	C_7_H_6_O_5_	125.02 (M-H-CO_2_), 107.03 (125.02-H_2_O), 79.02
3	2.02	Catechinic acid	289.07	C_15_H_12_O_6_	245.03 (M-H-CO_2_), 217.04 (245.03-CO), 109.03
4	2.19	Mangiferin	421.08	C_19_H_18_O_11_	331.04 (M-H-C_6_H_8_O_4_), 301.03 (331.04-CO), 273.02
5	2.25	Hyperoside	463.09	C_21_H_20_O_12_	301.04 (M-H-C_6_H_10_O_5_), 273.05 (301.04-CO), 179.03
6	2.31	Kaempferol	285.04	C_15_H_10_O_6_	257.05 (M-H-CO), 185.03 (257.05-C_5_H_8_O), 153.02
7	2.92	Ellagic acid	300.99	C_14_H_6_O_8_	273.00 (M-H-CO), 245.01 (273.00-CO), 185.00
8	2.93	Quercetin	301.03	C_15_H_10_O_7_	273.05 (M-H-CO), 199.03 (273.05-C_5_H_8_O), 179.03
9	3.48	Protocatechuic acid	153.02	C_7_H_6_O_4_	109.03 (M-H-CO_2_), 91.04 (109.03-H_2_O), 73.03
10	11.45	Puerarin Puemfin	415.10	C_21_H_20_O_9_	253.05 (M-H-C_6_H_10_O_5_), 225.06 (253.05-CO), 121.03
11	13.14	p-Coumaric acid	163.03	C_9_H_8_O_3_	119.06 (M-H-CO_2_), 91.07 (119.06-C_2_H_2_), 73.05

## Data Availability

The original contributions presented in this study are included in the article. Further inquiries can be directed to the corresponding author.
